# CUMULATIVE ADVANTAGE IN CHANGING ECONOMIC TIMES: STRESS, DISTRESS, AND FUTURE PROSPECTS FOR AGING COHORTS

**DOI:** 10.1093/geroni/igz038.1559

**Published:** 2019-11-08

**Authors:** Dale Dannefer, Stephen Crystal, Angela O’Rand

**Affiliations:** 1 Department of Sociology, Case Western Reserve University, Cleveland, Ohio, United States; 2 Rutgers University, New Brunswick, New Jersey, United States; 3 Departnbet of Sociology, Duke University, Durhan, North Carolina, United States

## Abstract

Processes of cumulative dis/advantage operate within cohorts and across historical time. In the ongoing dance of age, cohort and period, each cohort encounters distinctive social and economic environments at particular ages that may ameliorate or exacerbate the cumulative and systemic processes of inequality production that operate over its collective life course. We explore issues of current and future late-life inequality and its consequences. As overall income inequality has grown, what are the likely consequences for late-life outcomes? How have cohorts currently in midlife been affected by the Great Recession of 2008 and subsequent recovery? What are the mental and physical consequences of these developments, and to what extent can they be ameliorated by interventions in middle and later adulthood? This symposium addresses how variation in economic circumstances and social and psychological stresses may affect outcomes over the life course, and how these complex, interacting processes can be best conceptualized and examined. One paper examines the impact of the Great Recession and subsequent events on the intracohort distribution of income, suggesting inordinate setbacks during the Recession with likely long-term effects for economically vulnerable subpopulations. Another explores the role of psychosocial stressors in the process of cumulative dis/advantage, focusing on linkages between functional limitations and psychological well-being in later life, and how these linkages are amplified by diverse dimensions of disadvantage (e.g., education, employment; coping strategies; caregiving). A third paper examines the intergenerational dimensions of cumulative advantage processes. Finally, contrasting theoretical frameworks for apprehending life-course processes and historical change will be explored.

